# Cesarean sections in a secondary level care hospital of Cameroon: an analysis of their six-year trends and adverse neonatal outcomes

**DOI:** 10.1186/s13104-017-2750-2

**Published:** 2017-08-29

**Authors:** Tsi Njim, Simeon-Pierre Choukem, Robinson Mbu

**Affiliations:** 1 Department of Internal Medicine and Pediatrics, Faculty of Health Sciences, University of Buea, Buea, Cameroon; 2 Health and Human Development (2HD) Research Network, Douala, Cameroon; 3 Centre for Tropical Medicine and Global Health, Nuffield Department of Medicine, University of Oxford, Oxfordshire, UK; 4 Department of Internal Medicine, Douala General Hospital, P.O. Box 4856, Douala, Cameroon; 5 Department of Obstetrics and Gynecology, Faculty of Medicine and Biomedical Sciences, University of Yaounde 1, Yaounde, Cameroon

**Keywords:** Cesarean section, Vaginal delivery, Instrumental vaginal delivery, Cameroon

## Abstract

**Background:**

The objectives of this study were to determine the trends of CS in a regional hospital in Cameroon and to explore its association with adverse neonatal outcomes.

**Methods:**

The study was conducted in the Buea Regional Hospital (BRH), Cameroon. A 6-year retrospective records analysis was used to determine the trends in rates of CS and neonatal adverse outcomes. In a 3-month prospective phase, indications of CS were identified.

**Results:**

Of a total of 4941 records reviewed from the year 2007 to 2012, the overall CS rate was 20.4%. The rates increased from 17.1% in 2007 to 20.9% in 2012, with a peak of 22.7% in 2011, but this time-trend was not significant (p-trend =0.06). Three of the 25 cases of CS (12%) in the prospective phase were done at the request of mothers. The odds of having a low first minute APGAR (APGAR <7) in neonates born from CS were higher than in neonates born from a normal delivery (OR = 6.6 and 95% CI 5.7–7.7; p < 0.01).

**Conclusion:**

One out of every five women give birth through a CS in the BRH. This rate of CS is relatively high for a suburban population in a developing country. Strategies to reduce these rates should be investigated and instituted in the BRH to reduce health expenditures.

## Background

A cesarean section (CS) is an operative delivery through the anterior abdominal wall and uterus, usually performed when a vaginal delivery (VD) might put the life of the mother and/or her baby in danger [1, 2]. Reported rates of CS in developed countries vary from 6.2 to 36% and are increasing over time [3]. Developing countries are also joining this trend. The rate of CS in a Nigerian teaching hospital rose from 7.2% in 2000 to 11.8% in 2009 [4]. Several authors have expressed concern about these trends and the liberal approach taken by most clinicians towards CS [5, 6]; especially as the World Health Organization (WHO) proposed that the required CS rates in any region of the world should fall within the range of 10–15% [7].

The increasing rate of CS in developing countries constitutes a heavy economic burden because most of the CS performed do not have justifiable obstetric indications [8]. Women increasingly request a CS as opposed to a VD because of its perceived safety [9]. However, CS is associated with significant risk of morbidity and mortality to the mother as it is a major abdominal surgery [3]; women who give birth through CS are more likely to experience post-partum hemorrhage, thrombo-embolic complications, infections and prolonged hospital stay, as compared to those who deliver by vaginal route [9]. CS are therefore not risk-free.

In this study, we sought to determine the trend of CS rate over 6 years in a suburban hospital in Buea, and to identify if it has potential risks to the neonate.

## Methods

### Study design and setting

A 6-year retrospective register analysis was carried out to determine the rate, the yearly trends and to explore the associations with adverse neonatal outcomes of CS in the BRH. To provide data on indications of CS that were absent in registers, a 3-month prospective study was also performed.

The BRH is found in Buea with a total population of 90,088 inhabitants. It serves as a reference hospital in the South–West region of Cameroon, performing more deliveries than the other health facilities in the region. It also receives intrapartum transfers from the surrounding health facilities. The hospital has all the standard units of general medicine. One obstetrician assisted by a general practitioner was running the maternity at the time of the study. The institution lacks a neonatal intensive care unit.

### Study population and participants

The methods and data collection procedure have been fully described in a prior study which aimed to provide cut-off values for low birth weight and high birth weight [10–12]. For the retrospective register analysis, all the records of pregnant women who gave birth during a 6-year period from the 1st January 2007 to the 31st December 2012 were reviewed. Records were excluded if the gestational age was <28 completed weeks, if it reported multiple gestations or had incomplete information [12]. Information was said to be complete if it contained: maternal age, marital status, gestational age, type of delivery, birth weight, sex of the newborn and APGAR score at the first minute. These records had been collected and stored in a handwritten register by the obstetrician, general practitioners, midwives and trained nurses in the maternity.

For the prospective phase, we included all pregnant women who delivered at the BRH from January 2 to March 23, 2013, and their newborns. We excluded women who delivered at a gestational age below 28 weeks, those who had multiple gestations and those who did not provide written consent to take part in the study.

### Data collection

Information collected in the retrospective register analysis included the following:

- Socio-demographic characteristics; maternal age and marital status.
- Obstetric and clinical characteristics; gestational age, type of delivery (vaginal deliveries (VD), instrumental vaginal deliveries (IVD) or CS), and the APGAR score at the first minute.

Data collected in the prospective phase were used to determine the indications of CS. Pregnant women who were about to put to birth had their labour monitored and their socio-demographic characteristics (including socioeconomic status, and level of education) collected. The type of delivery was also recorded and the indications for CS noted.

The mother was then approached and informed of the entire study and her consent requested. Only those who agreed to participate in the study were included after they signed the written inform consent form.

### Data analysis

Data were analyzed using *Epi Info version 3.5.4.* Means (standard deviation) were used to summarize continuous variables, and proportions and frequencies for categorical variables. Frequencies were compared using Fisher’s exact tests while the trend in the series of prevalence was established using the Mann–Kendall test. The statistical significance was set at p < 0.05.

### Ethical considerations

Ethical approval was obtained from the Institutional Review Board of the Faculty of Health Sciences, University of Buea. The study also received administrative clearance from the South–West Regional Delegation of Public Health and from the Director of the BRH. For the prospective phase, informed consent was requested from the mothers after delivery and only those who agreed to participate in the study were included after they signed the inform consent form.

## Results

### Socio-demographic and obstetrical characteristics of mothers

In the retrospective phase, we reviewed a total of 4941 birth records from 6001 deliveries that occurred during the study period; with 649 records excluded due to missing information and 414 records excluded due to multiple gestations, babies born before arrival and abortions [12]. The mean age of the mothers was 26.4 ± 5.5 years. A majority (89.9%) had given birth at term, and 31.7% of them were single. Two hundred mother-newborn pairs were included in the prospective phase; their mean age was 26.4 ± 5.8 years. Also, 33 of the 200 cases (17.0%) were referred from other health care facilities and nine of these referred cases resulted in a CS.

### Yearly trend in rates of caesarean section

**Fig. 1:**
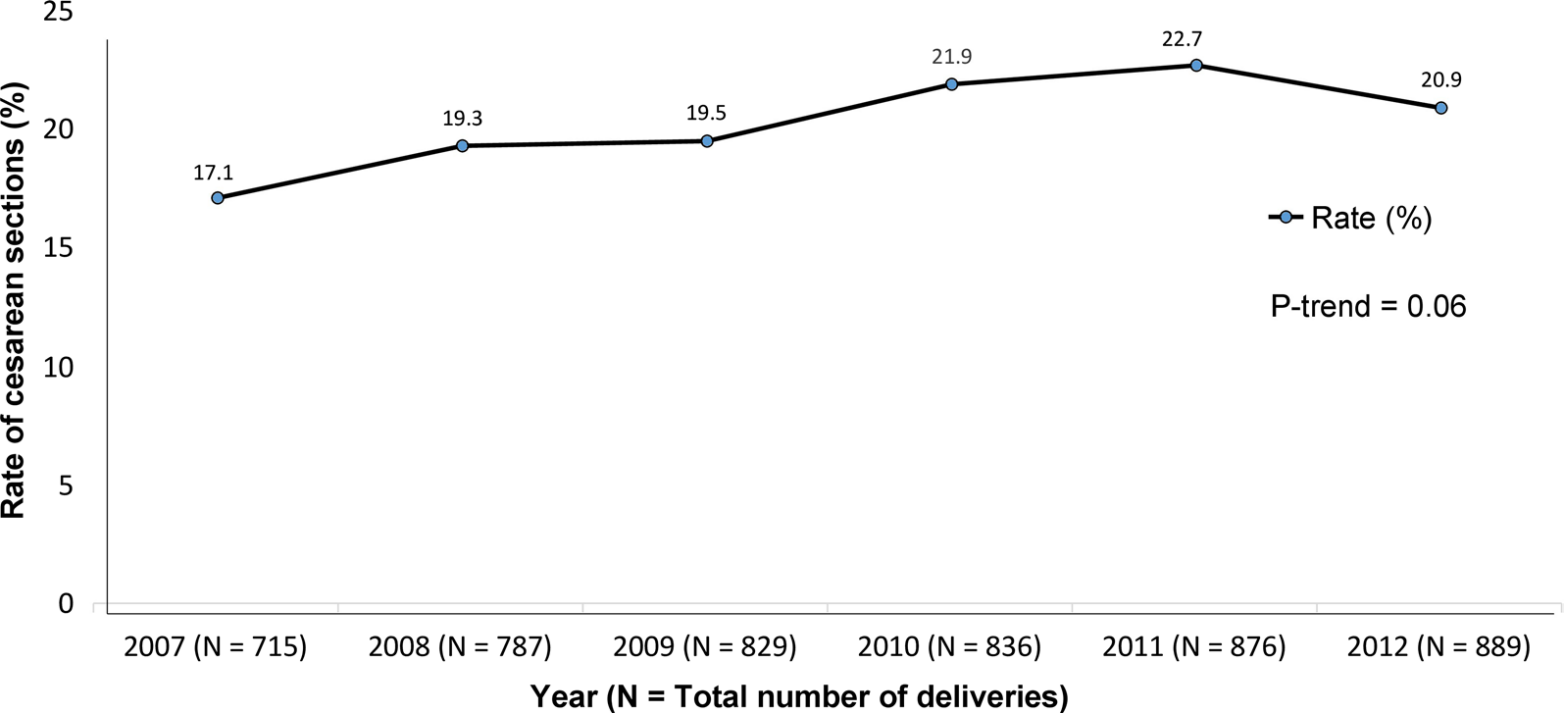
Rates of caesarean section in the Buea Regional Hospital from 2007 to 2012. The x-axis gives the various years or data collection. The y-axis represents the rate of CS for each given year. N represents the total number of deliveries that occurred each year. The *black line* links the various yearly rates of CS

Out of the 4941 mothers in the retrospective phase, 1006 had delivered through CS giving an overall rate of 20.4%. The rate of CS increased from 2007 (17.1%) to 20.9% in 2012, with a peak of 22.7% in 2011 (Fig. 1), but the time-trend over the 6 years was not significant (p = 0.06).

### Indications of cesarean section

Twenty-five of the 200 women (12.5%) in the prospective phase had a CS. The indications for CS were cephalopelvic disproportion (28%), acute fetal distress (28%), previous CS (16%), malpresentation (12%), maternal request (12%) and dystocia (4%).

### Adverse neonatal outcomes

The 1006 neonates delivered by CS were 3 times more likely to have an APGAR <7 at birth (56.7%) than neonates born through VD and IVD (16.6%) (p < 0.01). There was no significant difference in the frequency of stillbirths amongst women who gave birth through CS and VD or IVD (Table 1).

**Table 1:** Adverse neonatal outcomes of CS amongst the 4941 neonates

Complications	CS		VD + IVD		Odds ratio	95% CI	*p* value
	N	(%)	N	(%)			
APGAR <7 (1st min)							
Yes	572	56.7	654	16.6	6.6	5.7–7.7	<0.01
No	434	43.1	3281	83.4	1.0		
Stillbirth							
Yes	43	4.3	131	3.3	1.3	0.9–1.8	0.09
No	963	95.7	3804	96.7	1.0		

*CS* caesarean section, *IVD* instrumental vaginal delivery, *VD* vaginal delivery

## Discussion

In this study, we found that the overall rate of CS was 20.4%, there was an insignificant trend towards an increase of this rate over time. Seventeen percent of the participants were referred from surrounding health facilities and 27.3% of the referred cases resulted in a CS. CS was associated with a higher proportion of neonates with APGAR <7 at birth. We also observed that 3 of the 25 CS cases in the prospective phase (12%) were performed upon women’s request.

The rates of CS we report in this study are relatively higher than the Cameroon national estimates [13] and rates reported in other regions in the country [14]. They also outweigh the WHO recommended range of 10–15%, released in 1985 [15] and maintained in an updated report in 2015 [16]. In a previous study performed in a University hospital in Yaoundé the capital city of Cameroon, Nkwabong et al. found a rate of 12.7% in deliveries recorded from 1st January 2000 to 31 December 2004 [17]. This considerable difference in two health institutions in the same country cannot only be explained by an overall increase of the rate of CS over time in the country. It may reflect difference in attitudes towards elective CS without obstetrical indications or absence of uniform protocols and indications in the management of various obstetrical situations between hospitals of different levels. The national estimates of CS of 2.0% reported in 2012 [8] was not based on a scientific report, but on a 2004 survey by the Cameroon National Institute of Statistics, and may therefore not reflect the reality. Furthermore, we observed limited use of instrumental methods for VD in the BRH. Use of instrumental vaginal delivery could significantly reduce the rates of CS. Whatever the case, for a suburban population with limited socioeconomic resources, such a high rate of CS could represent a considerable economic burden.

Trends in CS rates are increasing in developing and developed countries with time [4, 18]. This may be in part due to the increased demand of some women to undergo elective CS without clear obstetrical indications. In our study, 12% of the CS in BRH were done at the request of women, without any obstetrical indication. In the United States, McCourt et al. estimated from a systemic review that the rate of maternal request for CS was 0.3–14% of all CS [19]. The rate found in our study though obtained from a small sample in the prospective phase falls within the upper limits of this range and may suggest that women in developing countries are beginning to perceive CS to be relatively risk-free and less strenuous than VD as in developed countries.

The risk of having first minute APGARs <7 in babies born by CS seemed higher compared with those from normal deliveries. However, as shown by the prospective phase, about 28% of CS are done due to acute foetal distress. There is therefore the need to investigate this association further in future studies. Also, the overall rate of babies born with APGAR <7 in the BRH was quite high emphasising the need for adequate neonatal resuscitative procedures in this hospital.

We acknowledge the following potential limitations: the study was conducted in only one health facility, thus the rate obtained does not necessarily reflect the rates in all sub-urban areas in Cameroon. Furthermore, as a retrospective study, a potential risk that some of the variables in the records (APGAR score and gestational ages) were not correctly filled exists; as the study assesses a 6-year period during which there were some changes in personnel. Also, due to the number of missing records, there is the potential for a selection bias which could affect the trend in the rates of CS. Finally, the indications of CS were obtained from a prospective phase. Correlations with the retrospective phase were therefore difficult to make.

Nevertheless, the overall high rates of CS in a suburban hospital calls for a need to investigate the accuracy of indications; this could lead to a revision of continuous medical education policy on deliveries in Cameroon.

## Conclusion

One out of every five deliveries from 2007 to 2012 in the BRH was by CS. This rate is higher than those obtained earlier in other hospitals of the country and the WHO recommended rates. Our results call for a need to update and harmonize the indications of CS in the country. Reduction of the high rates for CS in this suburban population may reduce the health expenditures related with mother–child health.

## Abbreviations

BRH: Buea Regional Hospital
CI: confidence interval
CS: cesarean section
IVD: instrumental vaginal delivery
OR: odd ratio
VD: vaginal delivery
WHO: World Health Organization
